# Missing Information from the Estrogen Receptor Puzzle: Where Are They Localized in Bull Reproductive Tissues and Spermatozoa?

**DOI:** 10.3390/cells9010183

**Published:** 2020-01-10

**Authors:** Jana Antalikova, Petra Secova, Lubica Horovska, Romana Krejcirova, Ondrej Simonik, Jana Jankovicova, Michaela Bartokova, Lucie Tumova, Pavla Manaskova-Postlerova

**Affiliations:** 1 Laboratory of Reproductive Physiology, Institute of Animal Biochemistry and Genetics, Centre of Biosciences, Slovak Academy of Sciences, 840 05 Bratislava, Slovakia; jana.antalikova@savba.sk (J.A.); petra.secova@savba.sk (P.S.); lubica.horovska@savba.sk (L.H.); jana.jankovicova@savba.sk (J.J.); michaela.bartokova@savba.sk (M.B.); 2 Department of Veterinary Sciences, Faculty of Agrobiology, Food and Natural Resources, Czech University of Life Sciences Prague, 165 00 Prague 6, Czech Republic; krejcirova@af.czu.cz (R.K.); simoniko@af.czu.cz (O.S.); tumovalucie@af.czu.cz (L.T.); 3 Laboratory of Reproductive Biology, Institute of Biotechnology CAS, v.v.i., BIOCEV, 252 50 Vestec, Czech Republic

**Keywords:** reproduction, steroid hormones, testes, epididymis, bovine, plasma membrane

## Abstract

Estrogens are steroid hormones that affect a wide range of physiological functions. The effect of estrogens on male reproductive tissues and sperm cells through specific receptors is essential for sperm development, maturation, and function. Although estrogen receptors (ERs) have been studied in several mammalian species, including humans, they have not yet been described in bull spermatozoa and reproductive tissues. In this study, we analyzed the presence of all types of ERs (ESR1, ESR2, and GPER1) in bull testicular and epididymal tissues and epididymal and ejaculated spermatozoa, and we characterize them here for the first time. We observed different localizations of each type of ER in the sperm head by immunofluorescent microscopy. Additionally, using a selected polyclonal antibody, we found that each type of ER in bull sperm extracts had two isoforms with different molecular masses. The detailed detection of ERs is a prerequisite not only for understanding the effect of estrogen on all reproductive events but also for further studying the negative effect of environmental estrogens (endocrine disruptors) on processes that lead to fertilization.

## 1. Introduction

Estrogens are steroid hormones that affect a wide range of functions, especially those in reproductive organs [1,2]. Although estrogens were traditionally considered to be female hormones, it is now clear that they also have an important role in the male reproductive tract (reviewed in [3,4]).

Estrogens influence spermatogenesis in the testis [5,6], the transport and maturation of sperm within extra-testicular regions (such as efferent ductules and the epididymis) [7,8], capacitation [9,10,11], and the acrosome reaction [12,13,14]. To understand the mechanism of action of these hormones, the detection of receptor molecules in target cells is crucial. The presence of both nuclear and membrane estrogen receptors (ERs) has been documented in the cytosol, nucleus, plasma membrane, endoplasmic reticulum, and Golgi apparatus [15,16,17,18,19]. Currently, three types of ERs are known. Two of these, estrogen receptors 1 and 2 (ESR1 and ESR2) [20,21], are classical nuclear receptors; the third is the transmembrane receptor known as GPR30 or GPER1 (G-protein coupled estrogen receptor) [22]. Classical estrogen receptors are mediators of genomic cell signaling [23]; however, data suggest that they may also be involved in a rapid non-genomic signaling pathway [24,25], similar to that of GPER1. Moreover, it has been suggested that crosstalk between GPER1 and ESR1/2 facilitates the estrogen-induced activation of the rapid signaling pathway [26,27]. Estrogen receptors have been detected in male reproductive tissues, germ cells, and spermatozoa in mice [27,28], rats [29,30], bank voles [31,32,33], stallions [34,35], and humans [36,37,38,39], and a large number of publications have analyzed ERs in pigs [40,41,42,43,44,45]. However, there is no information regarding the presence of ERs in bull reproductive tissues and spermatozoa. Therefore, the aim of this study was to supplement the literature with completely new data on all types of ERs in bull testicular and epididymal tissues and epididymal and ejaculated spermatozoa. Using various detection and fixation methods, different antibodies, and appropriate controls, we conducted repeated experiments on several individual animals (tissues) and pooled samples (spermatozoa). As a result, we detected estrogen receptors ESR1, ESR2, and GPER1 in a bull model, and they are characterized here for the first time.

## 2. Materials and Methods

All chemical reagents were obtained from Sigma Aldrich (St. Louis, MO, USA) unless otherwise noted.

### 2.1. Antibodies

The antibodies against ESR1 were rabbit polyclonal antibody HC-20 (against the C-terminus of the human protein) (sc-543, Santa Cruz Biotechnology, Inc. Heidelberg, Germany) and mouse monoclonal antibody MA1-310 (against the synthetic peptide within the DNA-binding domain of the human protein) (Thermo Fisher Scientific, IL, USA). The antibodies against ESR2 were rabbit polyclonal antibody H-150 (against amino acids 1–150 of the human protein) (sc-8974, Santa Cruz Biotechnology, Inc. Heidelberg, Germany) and mouse monoclonal antibody 6A12 MA1-23221 (against amino acids 1–153 of the human protein) (Thermo Fisher Scientific, IL, USA). The antibodies against GPER1 were rabbit polyclonal antibody K-19 (against the internal region of human GPR30) (sc-48524-R, Santa Cruz Biotechnology, Inc. Heidelberg, Germany) and rabbit polyclonal antibody H-300 (against amino acids 76–375 of human GPR30) (sc-134576, Santa Cruz Biotechnology, Inc. Heidelberg, Germany). The controls used in the experiments were rabbit IgG isotype control (Novus Biological, Centennial, CO, USA) and mouse IgG1/IgG2 isotype control (EXBIO, Vestec, Czech Republic).

### 2.2. Bull Tissues and Spermatozoa

#### 2.2.1. Tissues

The bull testes and epididymides from three adult animals (*Bos taurus*) were obtained from a local slaughterhouse (Mala Maca, Slovakia). The study was carried out according to the Council Directive 98/58/EC, Council Regulation (EC) No. 1099/2009, Regulation (EU) 2016/1012, Slovak National Council No. 39/2007 and guidelines of the Slovak legislation (directive 432/2012 Z. z.).Tissue segments were preserved by TissueTek (Sakura Finetek, Alphen aan den Rijn, NL) and frozen in liquid nitrogen. Subsequently, 5 µm frozen sections were cut using a Leica Cryocut 1800 cryostat (Leica Microsystems, Wetzlar, Germany), fixed for 5 min in a cold ethanol–acetone mixture (1:1), air-dried, and washed in phosphate-buffered saline (PBS; 137 mM NaCl, 2.7 mM, 10 mM Na_2_HPO_4_.12H_2_O, 20 mM KH_2_PO_4_, pH 7.4). For detection of nuclear receptors (ESR1 and ESR2), some dried tissue sections were incubated for 5 min with a solution to disintegrate the nucleus (0.1 mM DTT, 2% Triton X-100, and 1000 IU heparin in PBS) at room temperature, washed twice with PBS, and air-dried.

#### 2.2.2. Ejaculated Spermatozoa

Freshly ejaculated or frozen-thawed spermatozoa from the three adult bulls (*Bos taurus*) used in experiments were obtained from Slovak Breeding Services, Inc. (Luzianky, Slovak Republic). Freshly ejaculated spermatozoa were separated from seminal plasma by centrifugation at 200× *g* for 10 min at room temperature and washed twice with PBS. Spermatozoa were resuspended in PBS to a final concentration of 10^8^ cells/mL. The pellets of cryo-conserved sperm were washed twice with PBS and centrifuged at 200× *g* for 10 min at room temperature. After washing, part of the spermatozoa suspension was fixed in 3.7% paraformaldehyde (PFD) in PBS for 10 min with stirring, washed two more times, and air-dried on slides. Another part of the spermatozoa suspension was applied on slides and fixed for 5 min by cold acetone–methanol (1:1) (wet fixation) and dried.

### 2.3. Collection of Spermatozoa from the Epididymis

The bull epididymis was dissected into three segments: the caput, corpus, and cauda. These tissue segments were used for the separation of epididymal spermatozoa. Each segment was cut into small pieces and incubated in 10 mL of PBS for 15 min at 37 °C; the cloudy suspension was then centrifuged at 50× *g* for 10 min to remove the tissue debris. For immunofluorescence analysis, spermatozoa were obtained after centrifugation at 200× *g* for 10 min and washed with PBS followed by centrifugation. Part of the spermatozoa suspension (10^8^ cells/mL) was fixed in 3.7% PFD in PBS for 10 min with stirring, washed two more times with PBS, and air-dried on slides. Another part of the sperm suspension was applied on slides and fixed for 5 min by cold acetone–methanol (1:1) (wet fixation) and dried. For detection of nuclear receptors (ESR1 and ESR2), some dried spermatozoa smears after fixations were incubated for 5 min with the nucleus-disintegrating solution at room temperature, washed twice with PBS, and air-dried.

### 2.4. In Vitro Spermatozoa Capacitation and Induction of the Acrosome Reaction

Freshly ejaculated spermatozoa were separated from seminal plasma by centrifugation at 200× *g* for 10 min at room temperature. For bovine sperm cell capacitation, washed spermatozoa were resuspended in a commercially supplied TL medium for bovine sperm capacitation (Minitube, Celadice, Slovak Republic) supplemented with 6 mg/mL bovine albumin serum, 0.02 M Na pyruvate, and 0.5 mg/mL gentamicin to a final concentration of 10^7^ cells/mL. Sperm cells were capacitated at 39 °C in 5% CO_2_ in a humidified atmosphere for 4 h. An acrosome reaction was subsequently induced by 10 µM Calcium Ionophore A23 187 (CaI) for 1 h at 39 °C in 5% CO_2_ in a humidified atmosphere.

### 2.5. Immunolabeling of Spermatozoa and Tissues

An immunofluorescence assay was performed on testicular and epididymal tissue sections and epididymal, freshly ejaculated, frozen-thawed, capacitated, and acrosome-reacted spermatozoa after blocking with Super Block^®^ Blocking Buffer (Thermo Scientific, Rockford, IL, USA) for 1 h at 37 °C. The tissue sections and sperm smears were treated with the appropriate primary antibody (anti-ESR1, anti-ESR2, or anti-GPER1) at a 1:100 dilution in PBS at a final concentration of 1–2 µg/mL. Goat anti-rabbit or horse anti-mouse IgG fluorescein (FITC)-conjugated secondary antibodies (Vector Laboratories, Burlingame, CA, USA) at a 1:300 dilution in saline were applied for 30 min in the dark at room temperature. The nuclear DNA of cells was stained by Vectashield mounting medium with DAPI (Vector Laboratories, Burlingame, CA, USA). The intactness of spermatozoa acrosomes was assessed by Rhodamine labeled Peanut Agglutinin (PNA-TRITC, Vector Laboratories Burlingame, CA, USA). All treatments were applied in a humidity chamber to prevent the cell smears and tissue sections from drying out. Rabbit IgG isotype control at the appropriate concentration (1–2 µg/mL) was applied as a control for primary polyclonal antibodies; IgG1 and IgG2 isotype controls were used for analyses with monoclonal antibodies. Immunostaining was evaluated under a Leica DM5500 B epifluorescence microscope at 400× and 1000× magnifications. The fluorescence images were recorded using a Leica DFC340 FX digital camera and processed using Leica Advanced Fluorescence software (Leica Microsystems, Wetzlar, Germany) or using a confocal scanning microscope and documented in ZEN lite software (Zeiss, Jena, Germany). Representative results are shown.

### 2.6. SDS-PAGE and Western Blot Analysis

The spermatozoa pellets were dissolved in reducing sample solutions (2% SDS in Tris–HCl buffer, pH 6.8, with 5% mercaptoethanol) with 0.5% Protease Inhibitor Cocktail, incubated for 30 min at 4 °C, and subsequently boiled for 5 min at 100 °C. Sperm protein extracts were separated by 12% SDS-PAGE and transferred onto nitrocellulose membrane (Advantec Toyo Kaisha Ltd., Tokyo, Japan). The molecular weights of the separated proteins were estimated using PageRuler Plus Prestained Protein Ladder (Thermo Scientific, Rockford, IL, USA) and Precision Plus Protein™ Dual Color Standards (Bio-Rad, Hercules, CA, USA). After blocking with 5% non-fat milk (SERVA Electrophoresis GmbH, Heidelberg, Germany) in T-PBS (0.1% Tween 20 in PBS), the membranes were incubated with primary antibodies anti-ESR1, anti-ESR2, anti-GPER1, controls (Rabbit IgG, Mouse IgG1/IgG2), and mouse monoclonal anti-α-tubulin antibody (DM1A; Sigma-Aldrich) overnight at 4 °C, followed by incubation with a secondary antibody: horse anti-mouse IgG/goat anti-rabbit IgG conjugated to horseradish peroxidase (HRP) (1:7500) (Vector Laboratories, Burlingame, CA, USA) or goat anti-mouse IgG/anti-rabbit IgG (whole molecule) conjugated to alkaline phosphatase for 1 h at room temperature. The antibody reaction was visualized with SuperSignal West Pico Chemiluminescent Substrate (Thermo Scientific, Rockford, IL, USA) for HRP-conjugated secondary antibodies or with NBT (4-nitroblue tetrazolium chloride) and BCIP (5-bromo-4-chloro-3-indolyl-phosphate) solution (MP Biomedicals, Santa Ana, USA) for secondary antibodies conjugated to alkaline phosphatase.

## 3. Results

### 3.1. Immunofluorescent Detection of Estrogen Receptors in the Bull Testes and Epididymis

**Figure 1:**
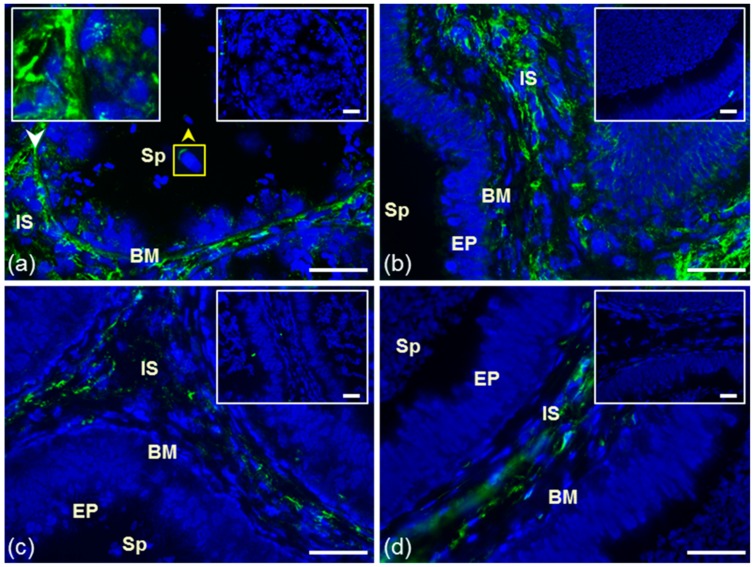
Reaction of anti-ESR2 antibody MA1-23221. Cryo-sections of bull reproduction tissues: testes (**a**) and epididymis: caput (**b**), corpus (**c**), and cauda (**d**). Tissues were treated with antibody MA1-23221 (green) or mouse IgG2 isotype control. Nuclear DNA was stained by DAPI (blue). Sp, spermatozoa; IS, interstitial tissue; BM, basal membrane; EP, epithelial cells. Controls are displayed in the top right corner of the figures. The white arrow points to the place depicted in the left frame, and the yellow arrow shows ESR2 localization in testicular sperm (**a**). Scale bar represents 50 µm.

The presence and distribution of estrogen receptor 1 (ESR1) were examined in cryo-sections of bull testes and the caput, corpus, and cauda epididymis using polyclonal antibody HC-20 and monoclonal antibody MA1-310. ESR1 detection in reproductive tissues probed by both antibodies was negative (Figure S1). Estrogen receptor 2 (ESR2) distribution was investigated by polyclonal antibody H-150 and monoclonal antibody MA1-23221. The signals of mAb MA1-23221 were observed in interstitial testicular tissue and in epithelium consisting of Sertoli and germ cells in various stages of development (Figure 1a). A weak signal was also observed in the interstitial tissue of the epididymis (Figure 1b–c). We did not detect ESR2 in either testicular or epididymal tissues when we used polyclonal antibody H-150. However, spermatozoa in sections of testicular and epididymal tissues treated with the nucleus-disintegrating solution were stained in the apical region of the acrosomal cap and neck with the H-150 antibody (Figure S2).

**Figure 2:**
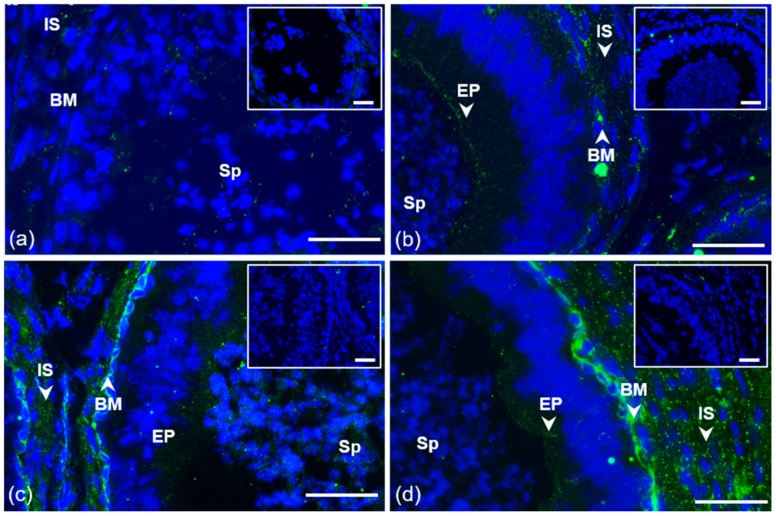
Localization of GPER1 in bull reproductive tissues. Cryo-sections of bull testes (**a**); caput (**b**), corpus (**c**), and cauda epididymis (**d**). Tissues were treated with antibody K-19 (green) or rabbit IgG isotype control. Sp, spermatozoa; IS, interstitial tissue; BM, basal membrane; EP, epithelial cells. Nuclear DNA was stained by DAPI (blue). Isotype controls are shown in the top right corner of the figures. Arrows show a positive reaction in tissues. Scale bar represents 50 µm (**a**–**d**).

Polyclonal K-19 and H-300 antibodies were used to detect the presence and distribution of GPER1 in cryo-sections of bull testes and epididymis. In the testicular tissues, the K-19 antibody did not react (Figure 2a). Signal of the K-19 antibody was observed in interstitial tissue (IS) of all epididymal parts and in the membrane of secretory epithelial cells (EP) in the caput and cauda epididymal tubule (Figure 2b–d). The reaction with polyclonal antibody H-300 in bull reproductive tissues was negative (Figure S3).

### 3.2. Immunofluorescent Localization of Estrogen Receptors in Bull Spermatozoa

The presence and distribution of ERs were examined in bull spermatozoa isolated from the epididymis (caput, corpus, and cauda) and in ejaculated (freshly ejaculated and cryo-conserved), in vitro capacitated, and acrosome-reacted spermatozoa after permeabilization with acetone–methanol.

**Figure 3:**
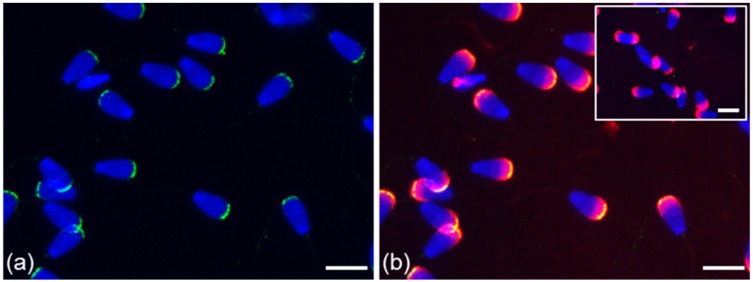
Localization of ESR1 in freshly ejaculated bull spermatozoa. Spermatozoa stained in the apical region of head with polyclonal antibody HC-20 (green) (**a**); spermatozoa stained with HC-20 (green), and sperm acrosomes labeled by PNA lectin (red) (**b**); nuclear DNA stained by DAPI (blue). Rabbit IgG isotype control is situated in the top right corner. Scale bar is 10 µm.

A strong specific signal of ESR1 detected by the HC-20 antibody appeared as a thin line in the apical part of the acrosome only in ejaculated spermatozoa, freshly ejaculated as well as frozen-thawed, that were permeabilized by acetone–methanol (Figure 3). Detection of ESR1 using monoclonal antibody MA1-310 was negative (Figure S4). The ESR1 pattern after the acrosome reaction differed between freshly ejaculated and frozen-thawed spermatozoa. In freshly ejaculated sperm, ESR1 localization detected by the HC-20 antibody remained unchanged after capacitation; the signal was lost after the acrosome reaction. In acrosome-reacted frozen-thawed spermatozoa, both HC-20 and MA1-310 antibodies detected ESR1, which was visible as in the equatorial or post-acrosomal region (Figure S5).

**Figure 4:**
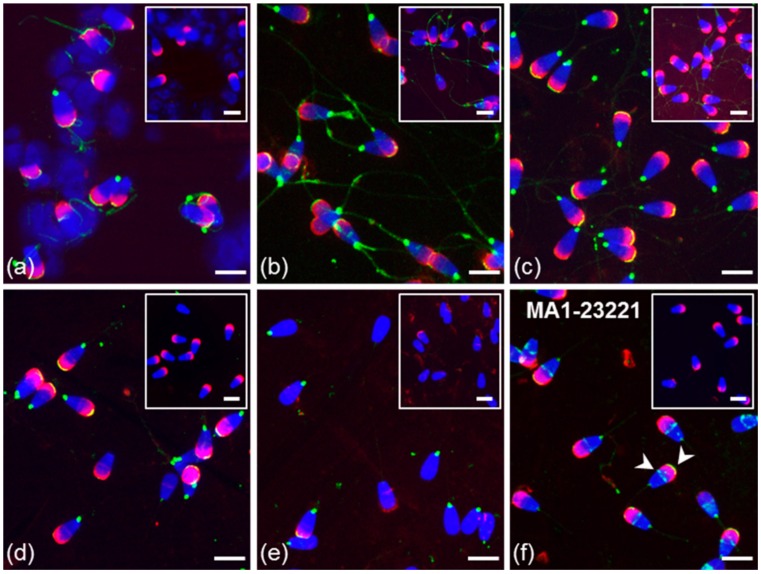
Localization of ESR2 in bull spermatozoa. Spermatozoa from testes (**a**) and the cauda epididymis (**b**); freshly ejaculated sperm (**c**,**f**); spermatozoa after in vitro capacitation (**d**); sperm after acrosome reaction (**e**). Spermatozoa were treated with polyclonal antibody H-150 (green) or rabbit IgG isotype control (**a**–**e**) or with monoclonal antibody MA1-23221 or mouse IgG2 isotype control (**f**). Arrows indicate the ESR2 detection in the apical and equatorial segment of sperm head with the MA1-2321 antibody (**f**). Nuclear DNA was stained by DAPI (blue), and spermatozoa acrosomes were labeled by PNA lectin (red). Controls are shown in the top right corner of the figures. Scale bar represents 10 µm.

In contrast to ESR1, ESR2 was localized in sperm cells in the lumen of testicular seminiferous tubules, and it remained visible in spermatozoa passing through the epididymis, as well as in ejaculated sperm (Figure 4a–c). A weak signal in the apical part of the sperm head detected by polyclonal H-150 antibody was amplified after treatment with the nuclear-disintegrating solution. An additional signal was observed in the neck of untreated sperm within the seminiferous tubule of the testis; in spermatozoa isolated from the epididymis; and freshly ejaculated and frozen-thawed spermatozoa. As detected by the H-150 antibody, the reaction pattern in the apical part of the acrosomal cap was unchanged after sperm capacitation and disappeared from spermatozoa after the acrosome reaction was induced (Figure 4d–e).

**Figure 5:**
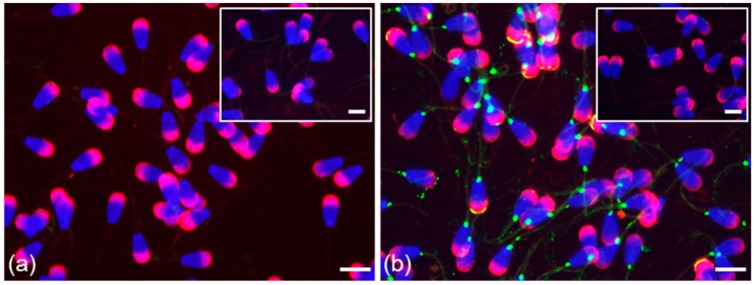
Localization of ESR1 and ESR2 in freshly ejaculated bull spermatozoa fixed in suspension with paraformaldehyde. Spermatozoa treated with anti-ESR1 antibody HC-20 (green) (**a**) without any positive reaction; spermatozoa treated with anti-ESR2 antibody H-150 (green) (**b**). Nuclear DNA was stained by DAPI (blue), and spermatozoa acrosomes were labeled by PNA lectin (red). Controls are displayed in the top right corner of the figures. Scale bar represents 10 µm.

The localization of ESR2 in the apical part of the acrosome was also confirmed by monoclonal antibody MA1-23221 in testicular spermatozoa (Figure 1a) and in ejaculated sperm; an additional signal appeared as a thin line in the equatorial segment area in the sperm subpopulation (Figure 4f; white arrow). In contrast to ESR1, the ESR2 signal of H-150 in the acrosomal cap and neck was also visible in the sperm suspension fixed by paraformaldehyde without permeabilization with acetone–methanol (Figure 5).

**Figure 6:**
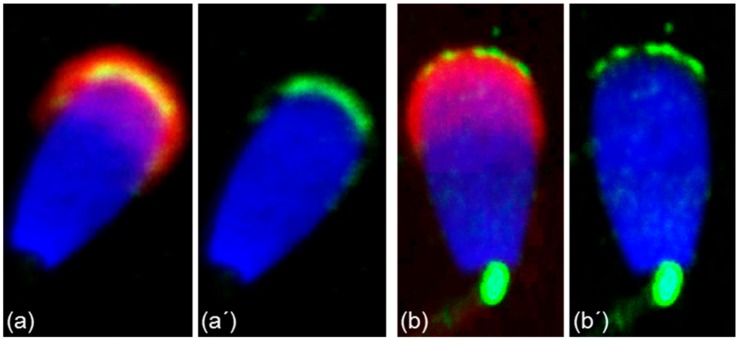
Different localization of ESR1 and ESR2 in freshly ejaculated bull spermatozoa shown by confocal microscopy. Spermatozoa treated with anti-ESR1 antibody HC-20 (green) (**a**,**a**’), spermatozoa treated with anti-ESR2 antibody H-150 (green) (**b**,**b**’). Nuclear DNA was stained by DAPI (blue), and sperm acrosomes were detected by PNA lectin (red).

More precise localization of the receptors in bull ejaculated spermatozoa was revealed by confocal microscopy. ESR1 was shown to be located in the acrosome cap, whereas ESR2 appeared to be localized in the apical ridge over the acrosomal membrane (Figure 6).

**Figure 7:**
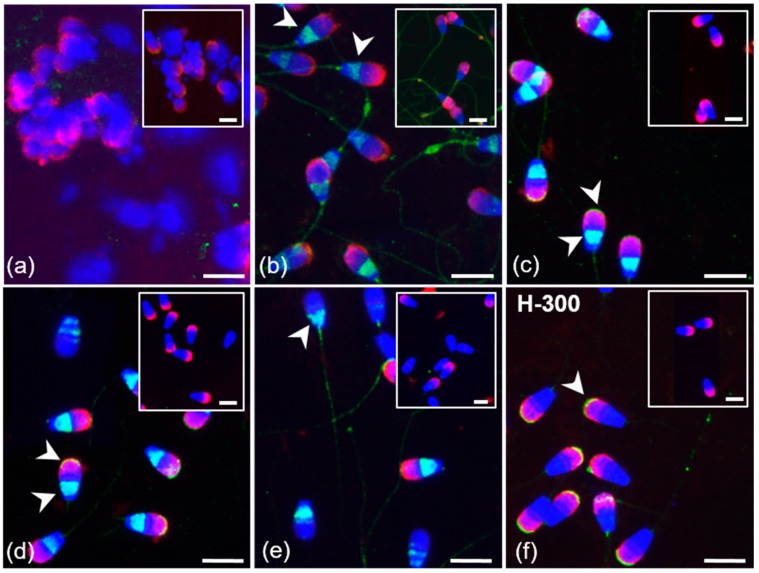
Localization of GPER1 in bull spermatozoa. Spermatozoa from testes (**a**) and the cauda epididymis (**b**); freshly ejaculated sperm (**c**,**f**); spermatozoa after in vitro capacitation (**d**); sperm after acrosome reaction (**e**). Spermatozoa were treated with polyclonal antibody K-19 (green) (**a**–**e**) or with polyclonal antibody H-300 (**f**) or rabbit IgG isotype control (**a**–**f**). Nuclear DNA was stained by DAPI (blue), and sperm acrosomes were labeled by PNA lectin (red). Isotype controls are shown in the top right corner of the figures. Arrows show a positive reaction in the equatorial and post-acrosomal region or apical acrosomal part of sperm head. Scale bar represents 10 µm.

Polyclonal K-19 and H-300 antibodies were used to detect the presence and distribution of GPER1 in sperm isolated from the epididymis, and ejaculated (freshly ejaculated and cryo-conserved), in vitro capacitated and acrosome-reacted spermatozoa. The K-19 antibody detected GPER1 in epididymal spermatozoa in the equatorial and post-acrosomal region (Figure 7b). Staining of the flagellum in cauda epididymal sperm was also observed in the isotype control treatment. In a subpopulation of ejaculated sperm (fresh and frozen-thawed), the K-19 antibody stained the equatorial or post-acrosomal region; an additional positive signal was observed in the apical part of the acrosome (Figure 7c). This localization of GPER1 in the apical region of the acrosomal cap was also detected in the majority of ejaculated spermatozoa by the H-300 antibody (Figure 7f). The reaction pattern of the K-19 antibody in the acrosomal part and post-acrosomal region remained unchanged after sperm capacitation. The signal in the apical part of the sperm acrosomal cap was lost after the acrosome reaction. The signal detected in the post-acrosomal region was still visible in part of the sperm population after the induction of the acrosome reaction (Figure 7e). The GPER1 staining of spermatozoa inside the seminiferous tubule was negative (Figure 7a).

### 3.3. Summarized Results of ER Localization in Bull Reproductive Tissues and Spermatozoa

**Figure 8:**
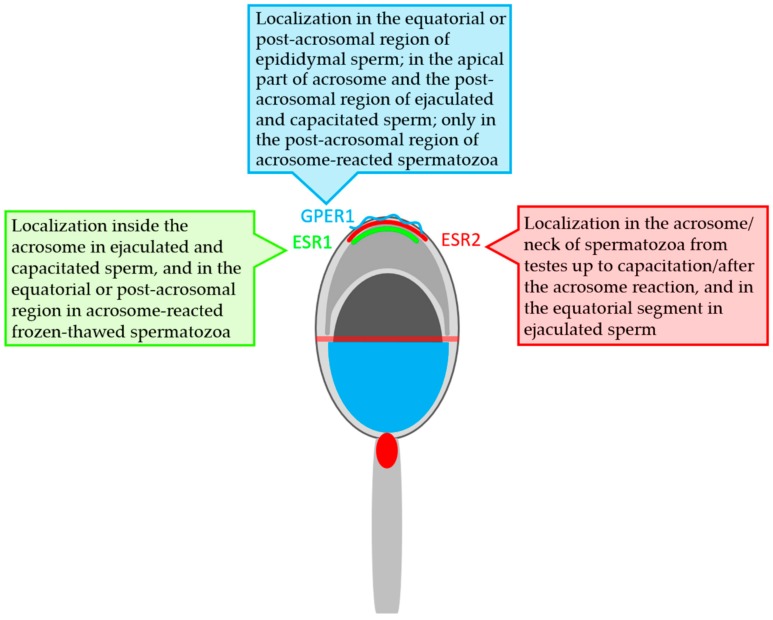
Graphical scheme of localization of the estrogen receptors in bull spermatozoa.

Our results from immunofluorescent study are summarized in Table 1 and graphically presented in the Figure 8 ESR1 was not found in either bull testes or epididymal tissues and spermatozoa. ESR1 was detected inside the acrosome in ejaculated and capacitated spermatozoa. On the other hand, ESR2 was detected in the acrosome and neck of spermatozoa at different stages, from testes to capacitated sperm. ESR2 is probably localized close to the plasma membrane in the acrosomal cap. Additionally, this receptor was found in the testicular and epididymal tissues of bulls. GPER1 was shown to occur in epididymal sperm, in which its signal was in the equatorial and post-acrosomal region. In bull ejaculated and capacitated spermatozoa, GPER1 was detected not only in the post-acrosomal region: an additional signal occurred in the apical part of the acrosome. The GPER1 signal in the post-acrosomal region was still detectable in acrosome-reacted spermatozoa. A positive reaction with GPER1 was observed in the secretory epithelium and interstitial tissue of bull epididymis.

### 3.4. Western Blot Immunodetection of Estrogen Receptors in Bull Sperm Extracts

**Figure 9:**
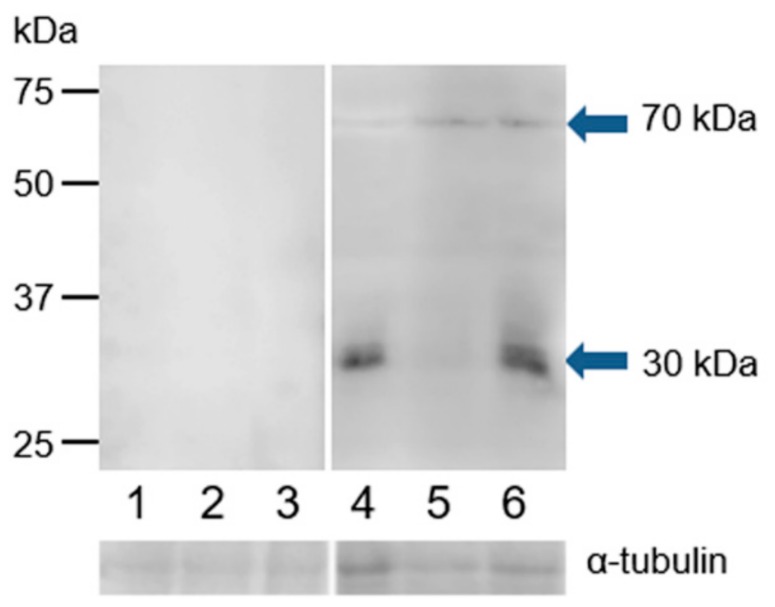
Reaction of anti-ESR1 (HC-20) with protein extracts from bull spermatozoa. Bull spermatozoa proteins were analyzed after separation by SDS-PAGE (12% gel) under reducing conditions followed by Western blotting with polyclonal antibody HC-20. 1, spermatozoa from the caput epididymis; 2, spermatozoa from the corpus epididymis; 3, spermatozoa from the cauda epididymis; 4, freshly ejaculated spermatozoa; 5, spermatozoa after in vitro capacitation; 6, spermatozoa after the acrosome reaction. The arrows indicate different protein isoforms of ESR1. The protein concentration in sperm samples was checked by α-tubulin detection.

We used monoclonal antibody MA1-310 and polyclonal antibody HC-20 to investigate ESR1 protein in the extracts of bull spermatozoa isolated from three parts of the epididymis, ejaculated spermatozoa, and spermatozoa after in vitro capacitation and the acrosome reaction. The polyclonal antibody recognized two protein bands of approximately 30 and 70 kDa in the extracts of spermatozoa after ejaculation, capacitation, and the acrosome reaction. In the extract from capacitated sperm, the antibody showed a weaker reaction with the 30 kDa protein band (Figure 9). Monoclonal antibody MA1-310 did not detect any protein bands in sperm extracts (Figure S6).

**Figure 10:**
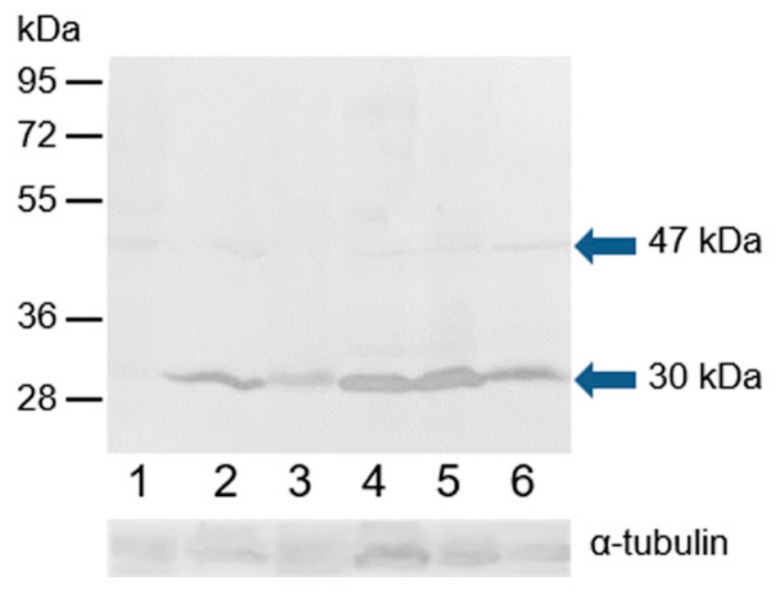
Reaction of anti-ESR2 (H-150) with proteins extracted from bull spermatozoa. Western blotting with antibody H-150 was performed to analyze bull spermatozoa proteins after protein separation by SDS-PAGE (12% gel) under reducing conditions. 1, spermatozoa from the caput epididymis; 2, spermatozoa from the corpus epididymis; 3, spermatozoa from the cauda epididymis; 4, freshly ejaculated spermatozoa; 5, spermatozoa after in vitro capacitation; 6, spermatozoa after the acrosome reaction. The arrows indicate different isoforms of ESR2. The protein concentration in sperm samples was checked by α-tubulin detection.

Monoclonal antibody MA1-23221 and polyclonal antibody H-150 were used to examine the ESR2 protein in the extracts of bull epididymal and ejaculated spermatozoa and spermatozoa after in vitro capacitation and the acrosome reaction. In the Western blot analysis under reducing conditions, polyclonal antibody H-150 strongly reacted with a band with a molecular mass of approximately 30 kDa, and an additional weak band of 47 kDa was detected in all sperm protein extracts (Figure 10). Monoclonal antibody MA1-23221 did not visibly react with sperm proteins (Figure S6).

**Figure 11:**
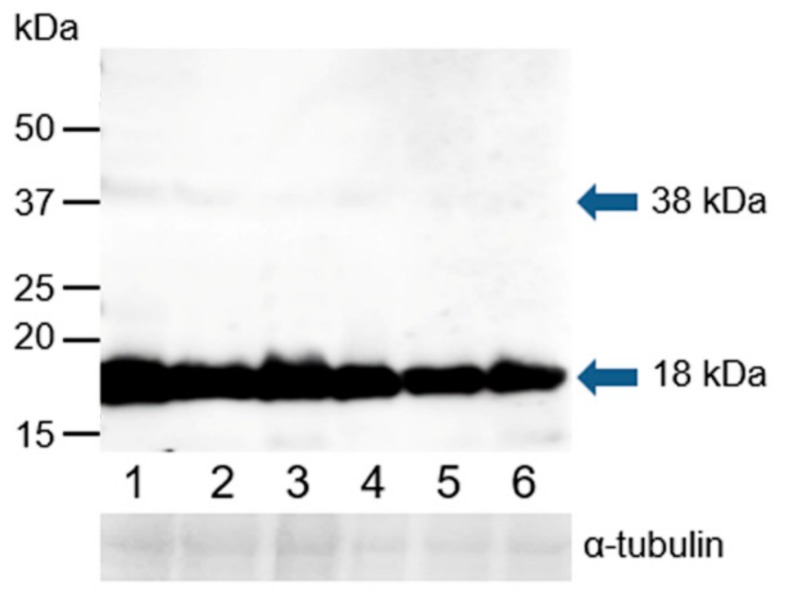
Reaction of anti-GPER1 (K-19) with proteins extracted from bull spermatozoa. Western blotting with antibody K-19 was performed to analyze bull spermatozoa proteins after their separation by SDS-PAGE (12% gel) under reducing conditions; 1, spermatozoa from the caput epididymis; 2, spermatozoa from the corpus epididymis; 3, spermatozoa from the cauda epididymis; 4, freshly ejaculated spermatozoa; 5, spermatozoa after in vitro capacitation; 6, spermatozoa after the acrosome reaction. The arrows indicate different protein isoforms of GPER1. The protein concentration in sperm samples was checked by α-tubulin detection.

Western blot analysis was performed under reducing conditions using K-19 and H-300 antibodies to detect GPER1 in the protein extracts of epididymal, ejaculated, and capacitated spermatozoa as well as spermatozoa after the induction of the acrosome reaction. The K-19 antibody detected one strong band with a molecular mass of 18 kDa, and after longer exposure time, an additional weak band of 38 kDa was visible in all analyzed sperm extracts (Figure 11). The H-300 antibody did not show any reaction with sperm proteins (Figure S7).

## 4. Discussion

The exact role of estrogen through ERs in the male reproductive tract is still under debate. Estrogens are produced in the seminiferous epithelium by the irreversible transformation of androgens by aromatase. In immature animals, the predominant source of aromatase is Sertoli cells; however, in the adult mammalian testis, aromatase is localized mostly in Leydig cells [46]. Estrogen receptors have distinct roles throughout the whole reproductive process. ERs in male reproductive tissues mediate sperm function during their testicular development [47] and epididymal maturation [2]. According to Dumassia et al. [48], ESR2 regulates spermatocyte apoptosis and spermiation, while ESR1 is mainly involved in spermiogenesis. Moreover, ESR1 is involved in regulating different epigenetic processes during spermatogenesis [49].

ESR1 and ESR2 have been found in the reproductive tract and the spermatozoa of many animal species, but published results on the detection and localization of both receptors are very diverse. Differences are caused by interspecies variance and the age of the studied animals (reviewed in [50]). Nevertheless, there is no available information on the presence of estrogen receptors in the male reproductive tract and spermatozoa in bulls.

In our study, we did not detect ESR1 in any cell types from bull testes or the epididymis, despite the use of two antibodies against different epitopes. Similarly, the absence of ESR1 in testes has also been reported in immature boars [42,51]; however, in mature boars, ESR1 has been detected in germ and Leydig cells [40,42]. In contrast to ESR1, ESR2 signals from the monoclonal anti-ESR2 antibody were observed in bull interstitial testicular tissue and in the epithelium comprising Sertoli cells and germ cells in various stages of development. Interestingly, in stallions, ESR2 was detected in Sertoli and Leydig cells of all animals, but ESR2 in germ cells was found only in pre-pubertal animals [35]. Similarly, Hess [2] reviewed the wider distribution of ESR2 relative to that of ESR1 in the male reproductive tract mainly in the interstitial tissue of the epididymis.

Our experiments showed that ESR1, in contrast to ESR2, was not detected in the bull epididymis. This fact suggests that ESR2 has a distinct role in the male reproductive tract of this mammalian species. In various mammals, there is evidence that ERs may play a crucial role in the reabsorption of testicular fluid from the rete testis, an event leading to the concentration of sperm before they enter the epididymal lumen. The structures responsible for this process are known as the efferent ductules [52]. In larger mammals, the efferent ductules are embedded entirely in connective tissue that is common to the head of the epididymis [53,54]. In ESR1 knock-out mouse males, this was associated with an increased frequency of damaged sperm membranes and abnormal sperm morphology linked to infertility. In contrast to ESR1-/- males, ESR2 knock-out mice were fertile [55].

Mature sperm cells are considered to be transcriptionally inactive but capable of translating synthesized mRNA [56]; therefore, it can be assumed that estrogens exert non-genomic rapid effects in spermatozoa [3]. In the female reproductive tract, ERs might have an effect on active transport of sperm to the site of fertilization [57]. ERs modulate the intracellular calcium level, which is a crucial factor of sperm capacitation [58]. Estrogens stimulate the progression of capacitation in boar spermatozoa in a concentration-dependent manner. Moreover, estrogens significantly increased the number of acrosome-reacted sperm after induction by zona pellucida [59].

In our analysis of bull spermatozoa, the presence and localization differed between ESR1 and ESR2. While ESR1 was visible only in the sperm after ejaculation, ESR2 was observed not only in ejaculated sperm but also in sperm from testes and the epididymis. These two classic ERs as transcription factors occur in the nucleus and may be present in the testicular germ cells [60]. However, we did not find them in the bull sperm nucleus even after the sperm treatment with the nuclear-disintegrating solution. Both receptors were detected in the acrosome of ejaculated spermatozoa after permeabilization of the plasma membrane, and ESR2 was additionally observed in the neck of sperm. The distinct results that showed both receptors in the acrosomal cap were obtained by polyclonal antibodies applied to sperm treated with paraformaldehyde (PFD). In contrast to ESR1, ESR2 was detectable in the apical ridge of the acrosomal membrane after PFD fixation, probably because the protein epitope was more accessible. The specific PNA labeling of the outer acrosomal membrane of bull sperm [61] indicates that PFD treatment caused the partial permeabilization of the plasma membrane. ESR1 is localized in a deeper layer of the acrosomal membrane oriented toward the lumen of the acrosome, accessible only after permeabilization by acetone–methanol.

Both the monoclonal and polyclonal antibodies used in the detection of ESR1 showed that its localization after the induction of the acrosome reaction differed in frozen-thawed and freshly ejaculated spermatozoa. The proportion of acrosome-reacted spermatozoa in freshly ejaculated and cryo-preserved sperm was similar (50–60%). However, in a certain sperm population after thawing, ESR1 was additionally localized in the equatorial and post-acrosomal region and remained present after the acrosome reaction. In the case of freshly ejaculated sperm, the induction of acrosomal exocytosis resulted in the loss of ESR1. The different behavior of ESR1 in the frozen-thawed sperm might be a consequence of mechanical and chemical stressors that alter the sperm surface [62]. Cryo-conservation of bull sperm causes irreversible changes in sperm structure, triggers signaling pathways leading to capacitation [63], and spermatozoa were characterized as capacitated or able to capacitate very easily (in 30 min) [64,65], so they might behave differently under capacitation conditions and thus after the induction of the acrosome reaction.

ESR2 antibody staining with H-150 showed a strong additional signal in the neck area of testicular, epididymal, and ejaculated spermatozoa. ESR2 localized in this area could reside in the redundant nuclear envelope (RNE) or centriole. The RNE, located between the plasma membrane and the flagellar base, has been confirmed to be a site of Ca^2+^ storage in bovine sperm [66]. The results of Fukami et al. [67] indicated that one of the functions of this structure is the progesterone-induced Ca^2+^ increase in mouse sperm. Similarly, the structure and/or function of centrioles might be influenced by steroid hormones [68]. The role of estrogens in the male reproductive tract is not restricted to sperm development and maturation; the differential localization of ESR1 and ESR2 suggests that they might have distinct roles in sperm function. The presence of ESR2 at different stages of spermatozoa (from those in testes to those after the acrosome reaction (centriole)) indicates the involvement of this receptor in the period from sperm development to fertilization and potentially after fertilization. The presence of ESR1 solely in spermatozoa after ejaculation, together with the presence of estrogens in bull seminal plasma [69], suggests the involvement of ESR1 in the physiological processes in sperm that lead to capacitation and possibly the acrosome reaction. The absence of ESR1 in the sperm of knock-out mice results in the increased frequency of spontaneous acrosome reactions [55]. In a bovine model, it was found that treatment with estradiol changed the kinetics of sperm release from oviductal epithelial cells induced by progesterone [70], thereby generally affecting the sperm fertilization ability.

Little information is available about the presence of GPER1 in reproductive tissue and spermatozoa, in contrast to the numerous studies that have focused on classical ERs. The only existing data regarding the expression of GPER1 in the epididymis were published recently for boars [46,71] and rats [72]. The presence of GPER1 in ejaculated sperm has only been reported in pigs, humans [42,73], and stallions [74,75]. Our analysis using the K-19 antibody revealed the presence of GPER1 in the epididymal interstitial and epithelial cells, as well as in spermatozoa isolated from the caput, corpus, and cauda. The signal in the equatorial or the post-acrosomal region that was detected in isolated epididymal sperm was not observed in sperm clumps within the lumen of tissue sections, probably because the epitope was poorly accessible to the antibody.

During the passage through the epididymis, the sperm surface is changed by the protein processing, removal and addition [76,77]. This is probably the reason why after ejaculation, an additional signal in the apical part of the acrosome was detected not only by the K-19 antibody but also H-300. While the majority of ejaculated spermatozoa were H-300 positive in the acrosome, K-19 labeled only a portion of the sperm population. This could be because the applied antibodies recognize different epitopes, or the plasma membrane was in a certain state.

The localization of GPER1 in the apical part of the acrosome of bull ejaculated spermatozoa suggests that this receptor plays a role in rapid signaling including ion fluxes (mostly calcium) [78] and secondary messengers that lead to kinase activity [79], such as the sperm capacitation and acrosome reaction. Thus, GPER1 is closely linked to both processes, involved in changes regarding calcium levels [73].

Our immunofluorescent data were confirmed by Western blot analysis of sperm protein extracts with polyclonal antibodies against all three ERs. Molecular weights of proteins recognized by the anti-ESR1 polyclonal antibody were approximately 30 and 70 kDa; the second one corresponding with the calculated molecular weight of bull ESR1 protein (66.5 kDa). Similarly, in an extract from porcine ejaculated spermatozoa, Rago et al. [41] detected this ER with a molecular mass of 67 kDa. The truncated 36 kDa isoform has been described in human uterine tissue and breast cancer cells [80]. The immunodetection of ESR2 in protein extracts confirmed the presence of this receptor in bull ejaculated and epididymal spermatozoa, which agrees with immunofluorescence results. The detected molecular masses of the ESR2 isoforms were 30 and 47 kDa. This is consistent with published data, where in bovine testis, the ESR2 truncated transcript of 1422 nucleotides has been detected at the mRNA level, which corresponds to a protein with an estimated molecular mass of 45 kDa. The other truncated transcript, ΔLBD isoform of ESR2 (30 kDa protein), was found [81]. Similarly, the mRNA of the ESR2 isoform from the human testicular cDNA library corresponds to a 28 kDa protein [82]. It has been proposed that the diversity of ESR2 isoforms implies a functional role of this phenomenon in the cellular physiological and pathological estrogen response [83]. Western blot analysis of protein extracts of epididymal and ejaculated sperm with anti-GPER1 antibody K-19 revealed the presence of bands with molecular masses of 18 and 38 kDa when the process of capacitation did not cause any change. The GPER1 band corresponding to a molecular mass of 38 kDa has also been detected in protein extracts of boar sperm from the cauda [46] and equine ejaculated spermatozoa [75]. Moreover, GPER was found as a 38 kDa protein in the cellular fraction of human testes [75,84]. Results of a study on the bovine genome [85] revealed a truncated isoform of G protein-coupled receptor 30 (354 AA). Furthermore, our detected low-molecular-mass isoform of 18 kDa corresponds to human GPER1 isoform 7, which has a calculated molecular weight of 19 kDa [86].

In recent years, new knowledge about the expression of classical ERs in human and pig spermatozoa has provided new insight into the relationship between estrogen action [55] and sperm development and function [47]. ESR1 and ESR2 were traditionally regarded as nuclear receptors that function as transcription factors. Then, Razandi et al. [87] reported that these ERs exist and function as plasma membrane receptors linked to G-protein [87]. The translocation of classical ERs to the plasma membrane is mediated by palmitoylation [16,79]. It has been suggested that the rapid signaling pathway is activated by estrogen through crosstalk between GPER1 and ESR1/2 [3,13,14]. The localization of ESR1, ESR2, and GPER1 within the acrosomal cap of bull spermatozoa seems to be a prerequisite for the possible co-operation between ERs involved in the events of the sperm lifetime.

## 5. Conclusions

In the presented work, we analyzed the presence of all types of ERs (ESR1, ESR2, and GPER1) in bull testicular and epididymal tissues and in epididymal and ejaculated spermatozoa for the first time. Additionally, we found two isoforms of each ER with different molecular masses: ESR1 (70 and 30 kDa), ESR2 (47 and 30 kDa), and GPER (38 and 18 kDa). The detailed detection of ERs is a prerequisite not only for understanding the influence of estrogens on all reproductive events but also for further studying the negative effect of endocrine disruptors (e.g., environmental estrogens, phytoestrogens, and xenoestrogens) on processes related to reproduction.

## Figures and Tables

**Table 1:** Immunofluorescent detection of ERs in bull reproductive tissues and spermatozoa with all used anti-ER antibodies.

Sample	Antibody					
	HC-20 (ESR1)	MA1-310 (ESR1)	H-150 (ESR2)	MA1-23221 (ESR2)	K-19 (GPER1)	H-300 (GPER1)
Testis	−	−	+	+	−	−
Caput Epididymis	−	−	+	+	+	−
Corpus Epididymis	−	−	+	+	+	−
Cauda Epididymis	−	−	+	+	+	−
Spermatozoa from the Caput Epididymis	−	−	+	−	+	−
Spermatozoa from the Corpus Epididymis	−	−	+	−	+	−
Spermatozoa from the Cauda Epididymis	−	−	+	−	+	−
Freshly Ejaculated Spermatozoa	+	−	+	+	+	+
Freshly Ejaculated Spermatozoa after in Vitro Capacitation	+	−	+	+	+	+
Freshly Ejaculated Spermatozoa after the Acrosome Reaction	−	−	±	−	+	−
Frozen-Thawed Spermatozoa	+	−	+	+	+	+
Frozen-Thawed Spermatozoa after in Vitro Capacitation	+	+	+	+	+	+
Frozen-Thawed Spermatozoa after the Acrosome Reaction	+	+	±	+	+	−

+ with reaction; − without reaction; ± reaction only in sperm neck.

## Supplementary Materials

The following are available online at https://www.mdpi.com/2073-4409/9/1/183/s1, Figure S1: Reaction of anti-ESR1 antibodies MA1-310 and HC-20 in bull reproductive tissues; Figure S2: Reaction of anti-ESR2 antibody H-150 in bull reproductive tissues; Figure S3: Reaction of GEPR1 (H-300) in bull reproductive tissues. Figure S4: Reaction of anti-ESR1 monoclonal antibody MA1-310 with freshly ejaculated bull spermatozoa. Figure S5: Localization of ESR1 with antibodies MA-310 and HC-20 in frozen-thawed bull spermatozoa after acrosome reaction. Figure S6: Reaction of anti-ESR1 (MA1-310)/anti-ESR2 (MA1-23221) with proteins extracted from bull spermatozoa. Figure S7: Reaction of anti-GPER1 (H-300) with proteins extracted from bull spermatozoa.
