# Supplementary material for: Missing Information from the Estrogen Receptor Puzzle: Where Are They Localized in Bull Reproductive Tissues and Spermatozoa?
Source: Cells. 2020 Jan 10;9(1):183. doi: 10.3390/cells9010183 (PMC7016540; doi:10.3390/cells9010183)
Supplement: Supplementary file 1 [file cells-09-00183-s001.zip › Supplements_revised2.docx]

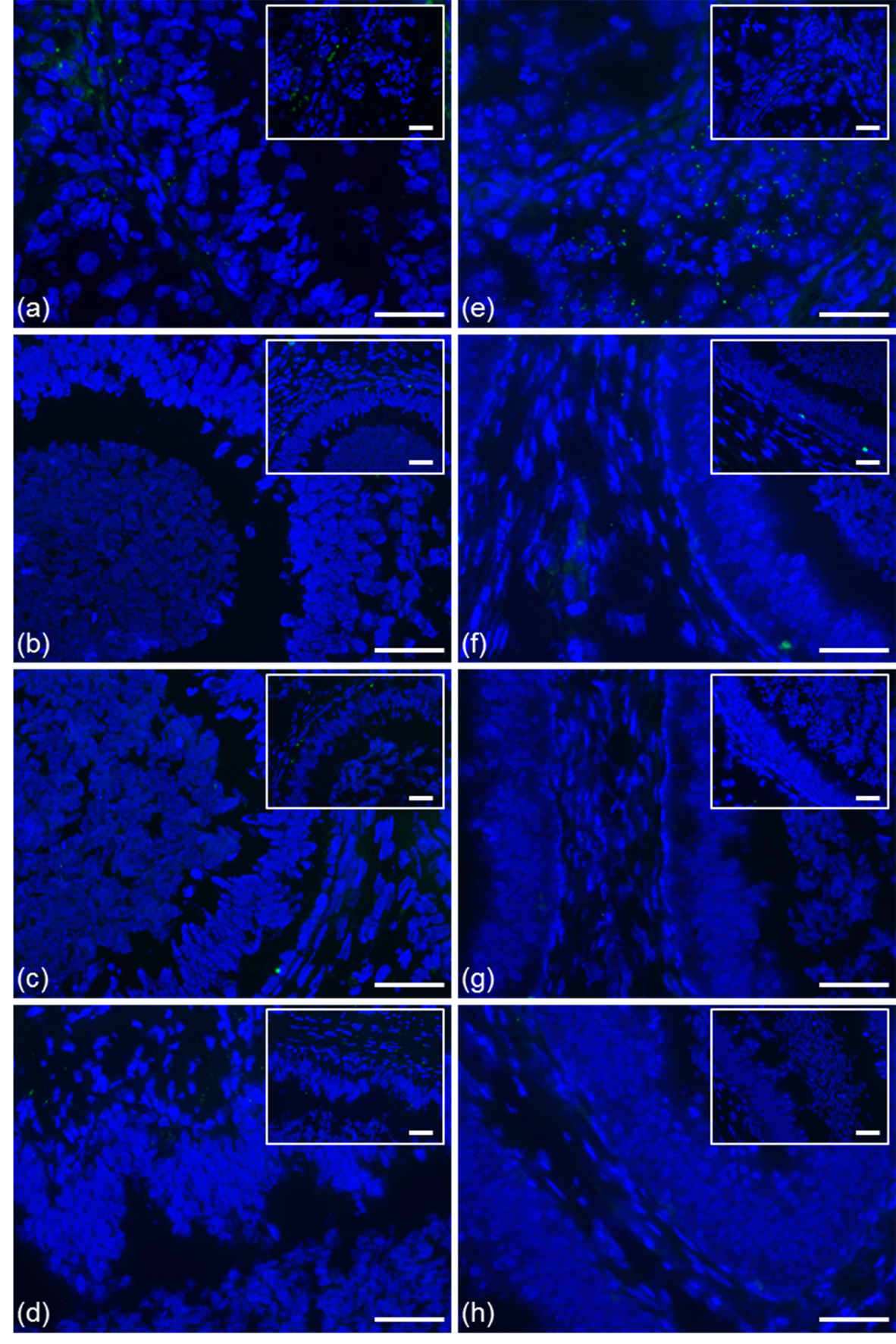


**Figure S1.** Reaction of anti-ESR1 antibodies MA1-310, HC-20 in bull reproductive tissues.Cryo-sections of bull reproduction tissues: testes (a, e) and epididymis: caput (b, f), corpus (c, g), and cauda (d, h). Tissues were treated with antibody MA1-310 (a-d) or HC-20 (e-h) without any positive reaction. Nuclear DNA was stained by DAPI (blue). Isotype controls are displayed in the top right corner of the figures. Scale bar 50 µm.


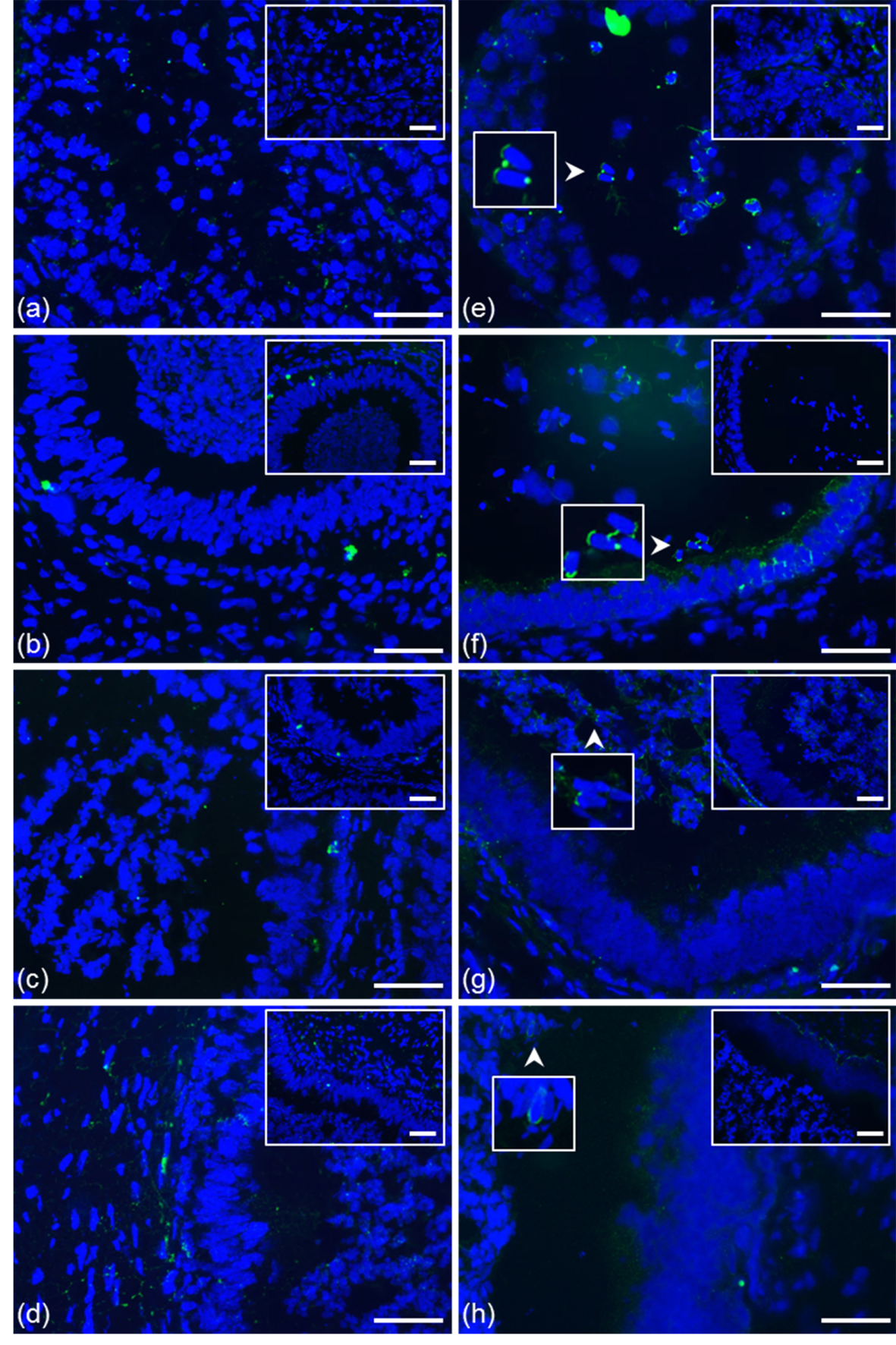


**Figure S2.** Reaction of anti-ESR2 antibody H-150 in bull reproductive tissues. Cryo-sections of bull testes (a, e) and epididymis: caput (b, f), corpus (c, g), and cauda (d, h). Tissues were treated with antibody H-150 (green) or rabbit IgG isotype control. Tissues (e-h) were treated with solution for disintegrating of nucleus before antibody treatment. Nuclear DNA was stained by DAPI (blue). Controls are displayed in the top right corner of the figures. Arrow points to the place depicted in the frame a positive reaction in sperm head (e-h). Scale bar 50 µm.

**Figure S3.** Reaction of GEPR1 (H-300) in bull reproductive tissues.Cryo-sections of bull testes (a) and epididymis: caput (b), corpus (c) and cauda (d). Tissues were treated with antibody H-300 without any positive reaction; nuclear DNA was stained by DAPI (blue); rabbit IgG isotype controls are displayed in the top right corner of the figures. Scale bar 50 µm.


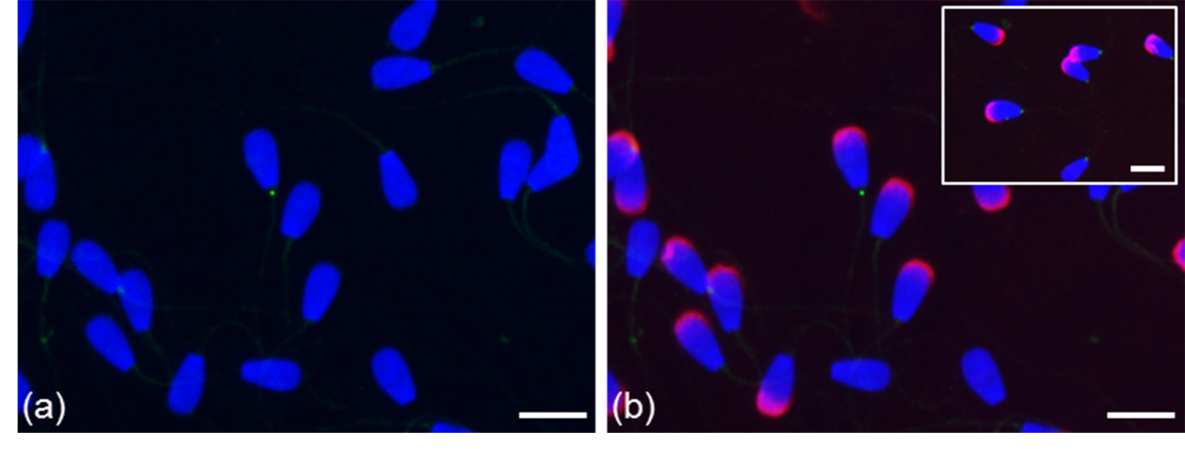


**Figure S4.** Reaction of anti-ESR1 monoclonal antibody MA1-310 with freshly ejaculated bull spermatozoa. Spermatozoa without antibody reaction (a); sperm acrosomes labelled by PNA lectin (red) (b); nuclear DNA stained by DAPI (blue). Mouse IgG1 isotype control is displayed in the top right corner of the figure. Scale bar 10 µm.


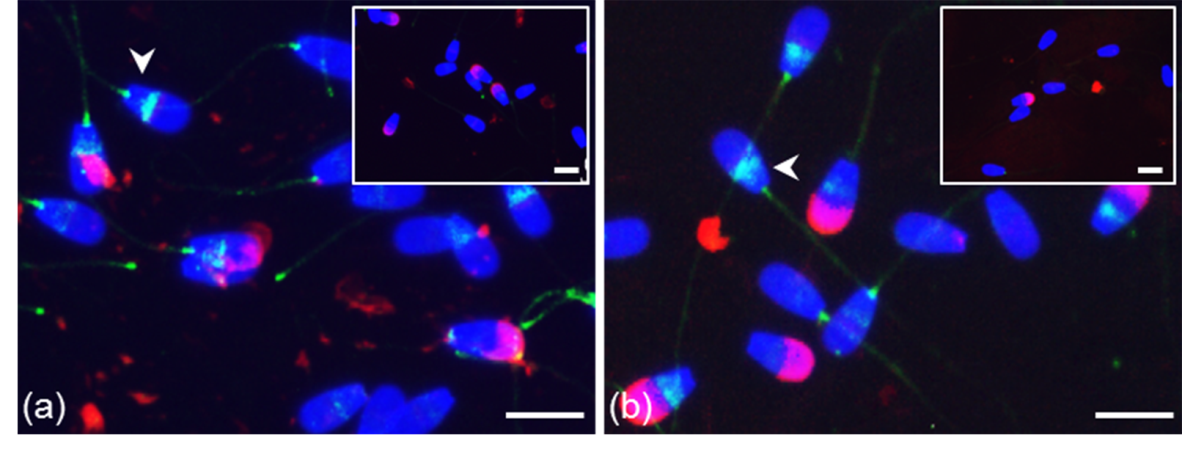


**Figure S5.** Localization of ESR1 with antibodies MA-310 and HC-20 in frozen-thawed bull spermatozoa after acrosome reaction.Spermatozoa stained with monoclonal antibody MA1-310 (green) (a); polyclonal antibody HC-20 (green) (b). Sperm acrosomes labelled by PNA lectin (red); nuclear DNA stained by DAPI (blue). Isotype controls are shown in the top corner of the figures; arrows show detection of ESR1 in the equatorial segment and post-acrosomal part. Scale bar 10 µm.


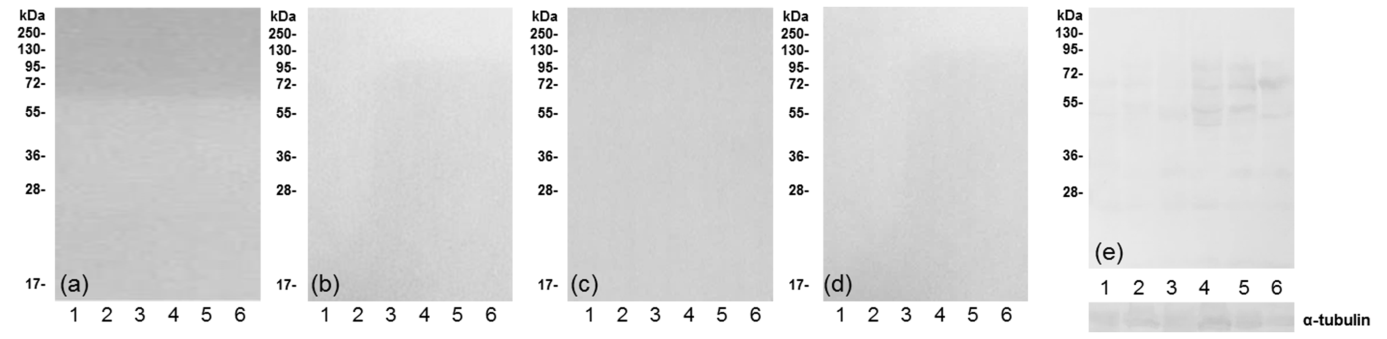


**Figure S6.** Reaction of anti-ESR1 (MA1-310)/anti-ESR2 (MA1-23221) with proteins extracted from bull spermatozoa.Western blotting with antibody MA1-310 (a), mouse IgG1 isotype control (b), monoclonal antibody MA1-23221 (c), mouse IgG2 isotype control (d) and rabbit IgG isotype control (e) was performed to analyse bull sperm proteins after protein separation by SDS-PAGE (12% gel) under reducing conditions. 1, spermatozoa from the caput epididymis; 2, spermatozoa from the corpus epididymis; 3, spermatozoa from the cauda epididymis; 4, freshly ejaculated spermatozoa; 5, spermatozoa after *in vitro* capacitation; 6, spermatozoa after the acrosome reaction. The protein concentration in sperm samples was checked by α-tubulin detection (e).


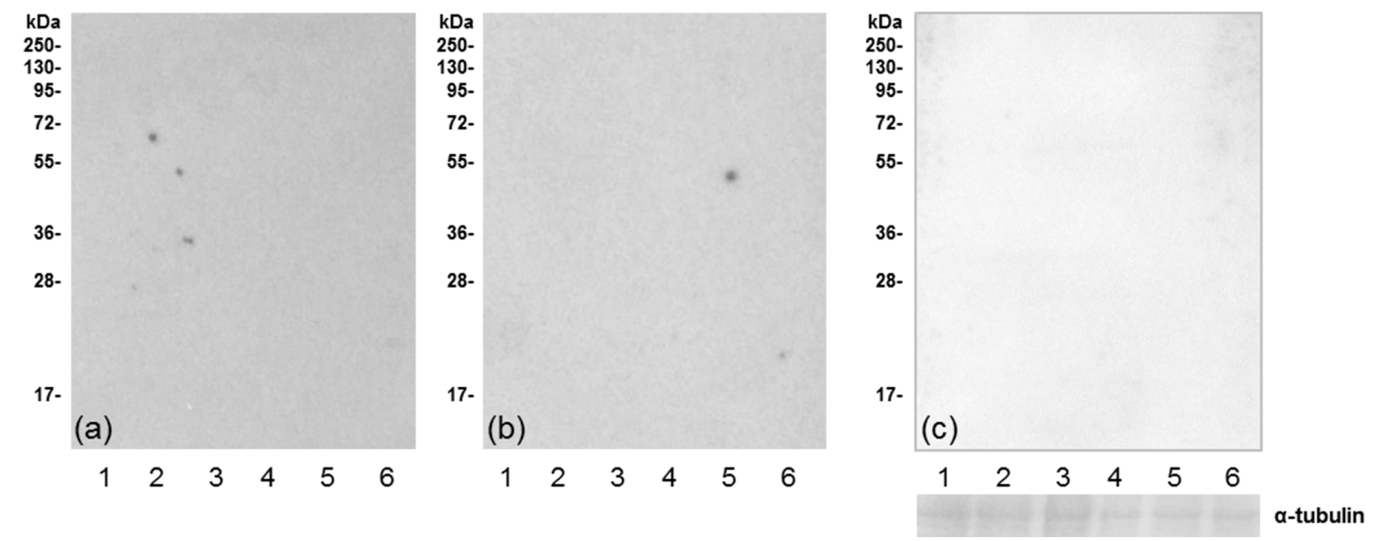


**Figure S7.** Reaction of anti-GPER1 (H-300) with proteins extracted from bull spermatozoa. Western blotting with antibody H-300 (a), rabbit IgG isotype control diluted 1:5000 (b) and rabbit IgG isotype control diluted 1:2500 (c) was performed to analyse bull sperm proteins after protein separation by SDS-PAGE (12% gel) under reducing conditions. 1, spermatozoa from the caput epididymis; 2, spermatozoa from the corpus epididymis; 3, spermatozoa from the cauda epididymis; 4, freshly ejaculated spermatozoa; 5, spermatozoa after *in vitro* capacitation 6, spermatozoa after acrosome reaction. The protein concentration in sperm samples was checked by α-tubulin detection (c).
