# Supplementary figures and images for: Missing Information from the Estrogen Receptor Puzzle: Where Are They Localized in Bull Reproductive Tissues and Spermatozoa?
Source: Cells. 2020 Jan 10;9(1):183. doi: 10.3390/cells9010183 (PMC7016540; doi:10.3390/cells9010183)

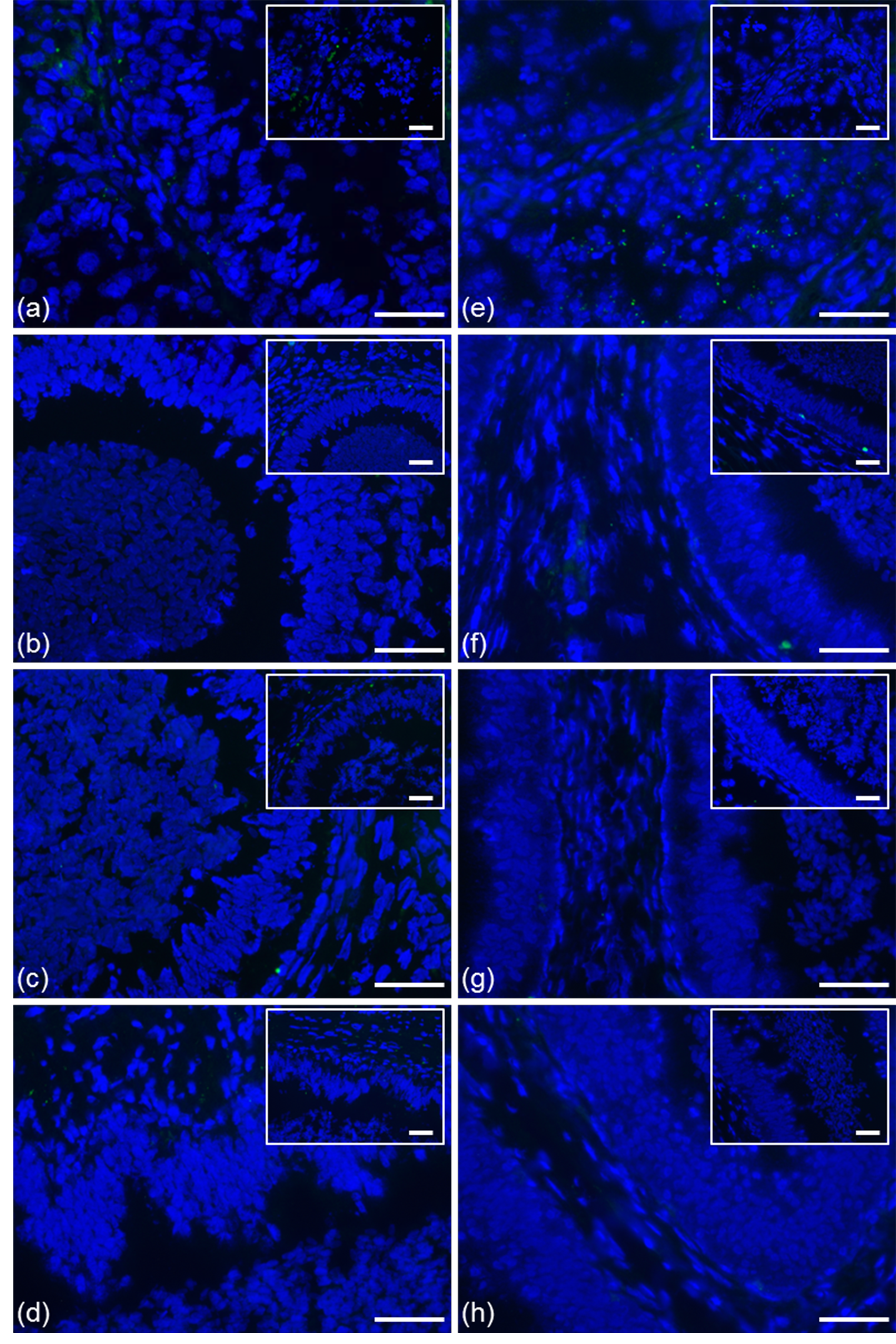

Supplement: Supplementary file 1 [file cells-09-00183-s001.zip › Supplement Figure S1.tif]

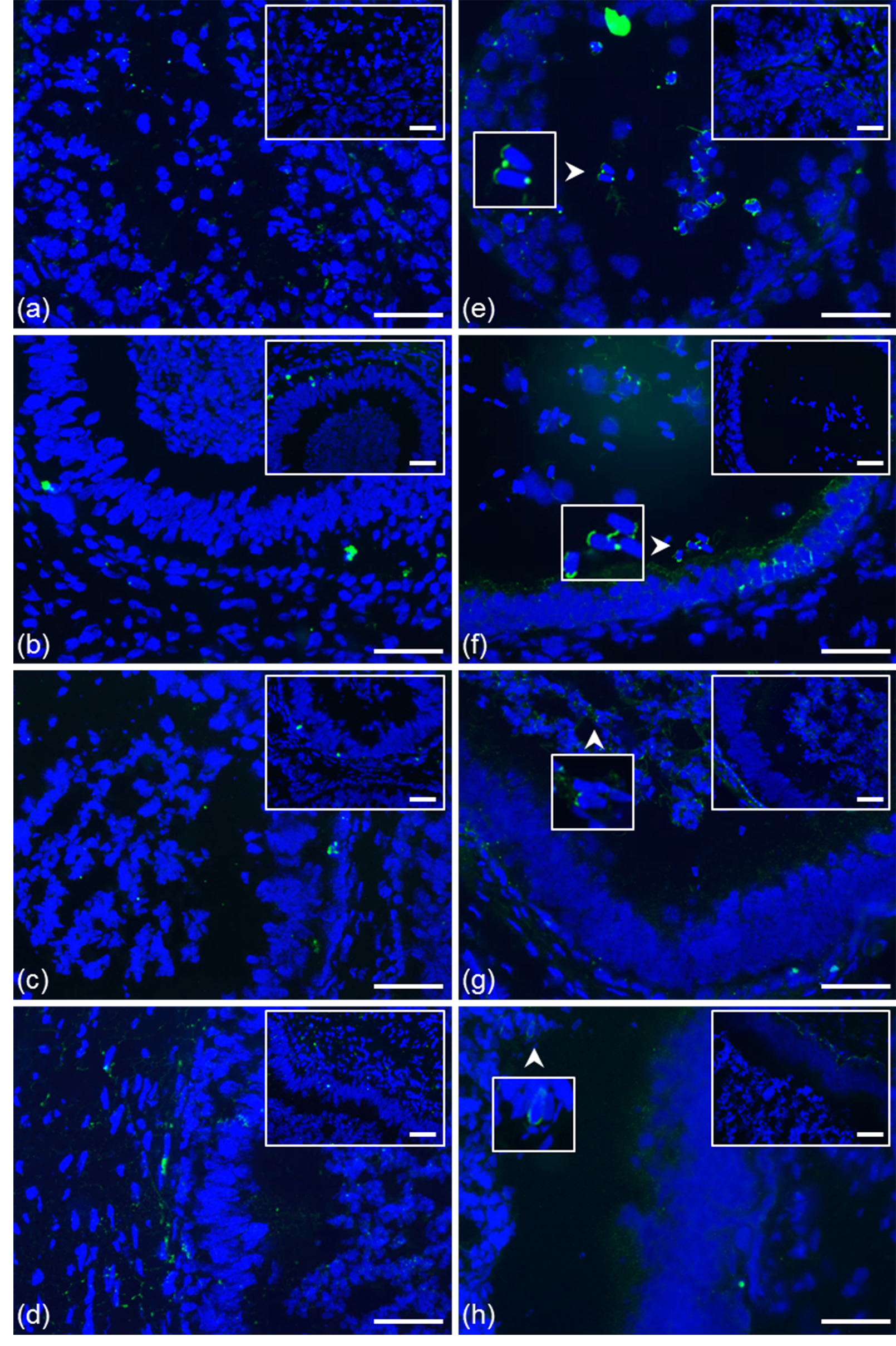

Supplement: Supplementary file 1 [file cells-09-00183-s001.zip › Supplement Figure S2.tif]

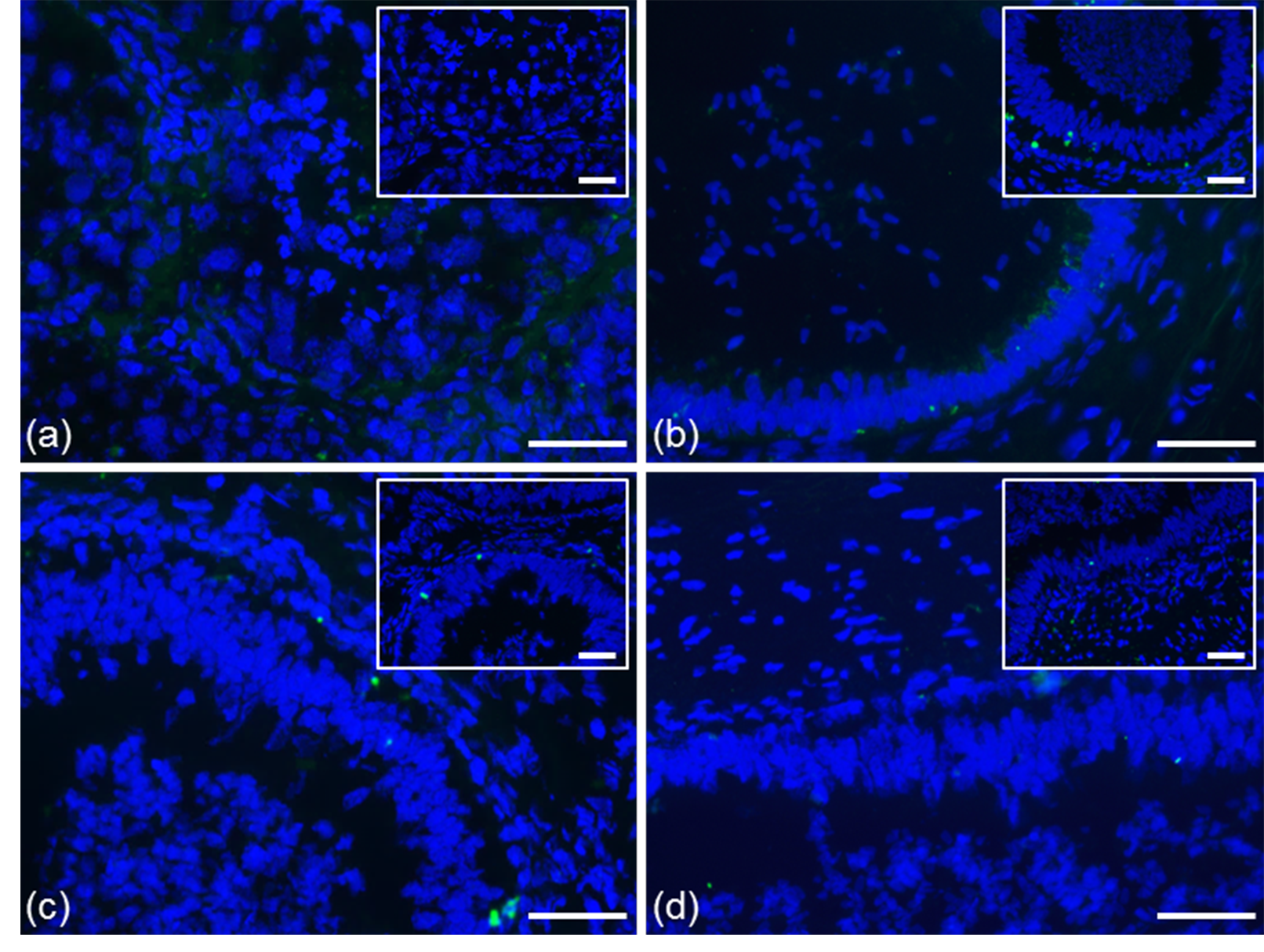

Supplement: Supplementary file 1 [file cells-09-00183-s001.zip › Supplement Figure S3.tif]

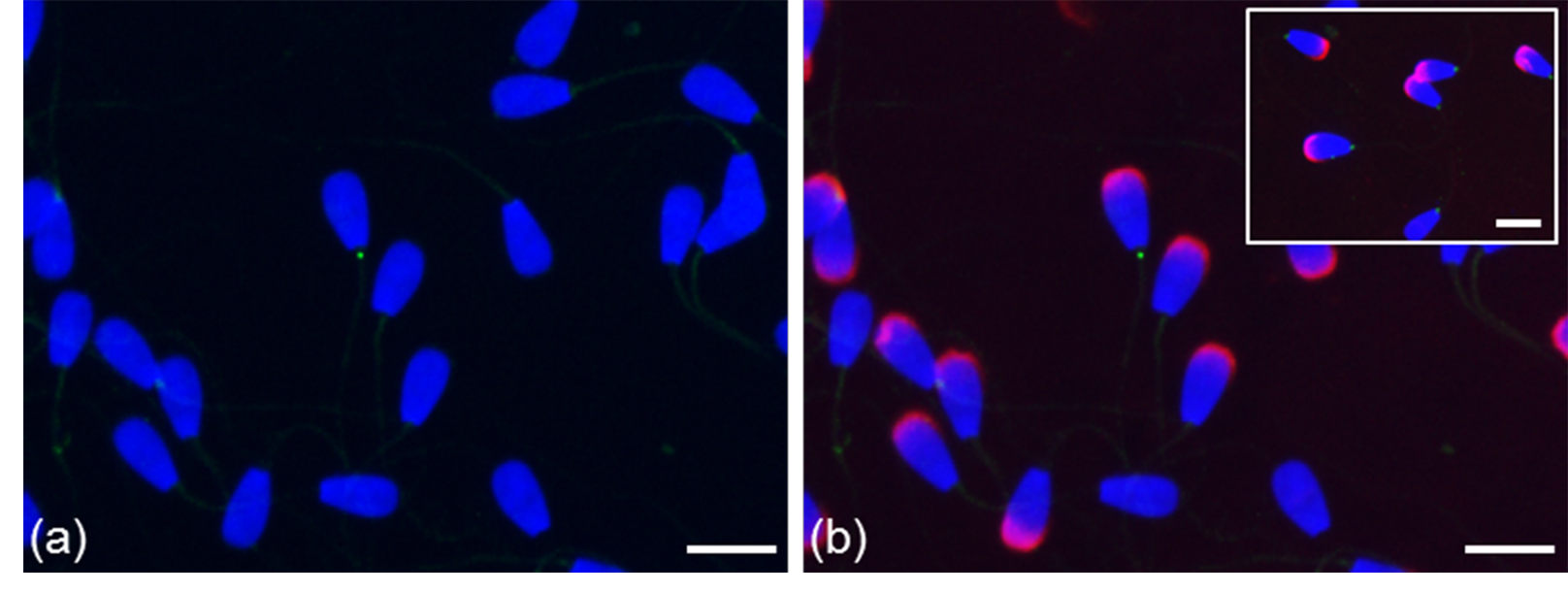

Supplement: Supplementary file 1 [file cells-09-00183-s001.zip › Supplement Figure S4.tif]

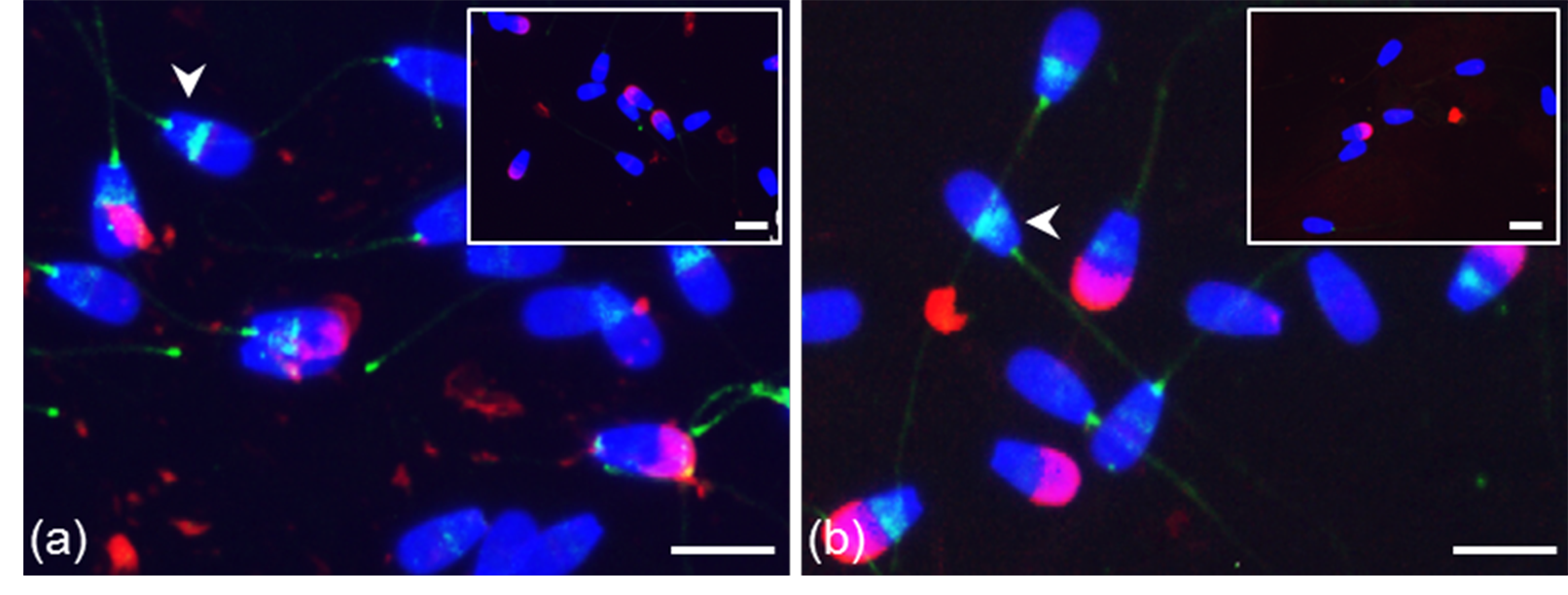

Supplement: Supplementary file 1 [file cells-09-00183-s001.zip › Supplement Figure S5.tif]

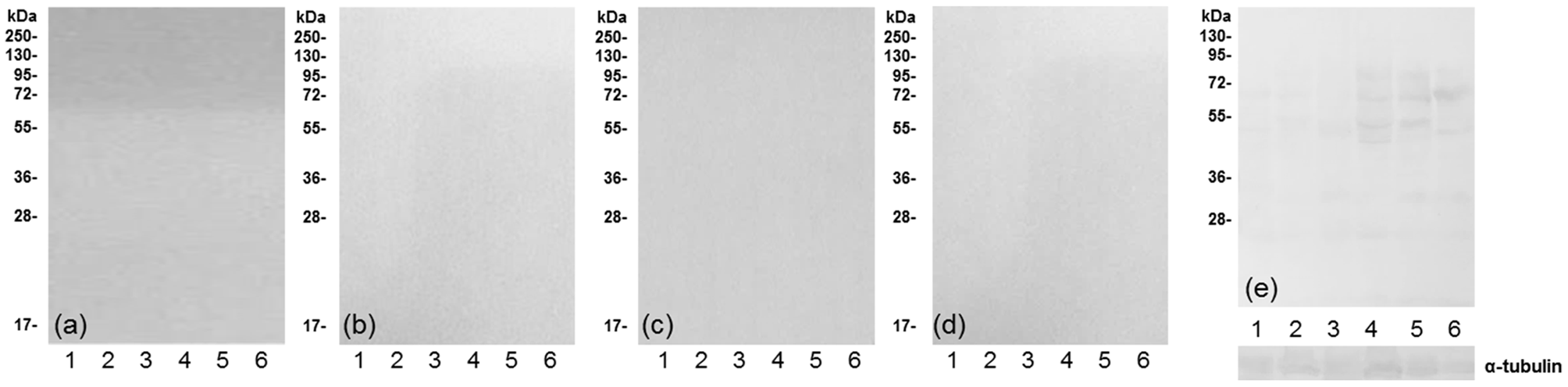

Supplement: Supplementary file 1 [file cells-09-00183-s001.zip › Supplement Figure S6.tif]

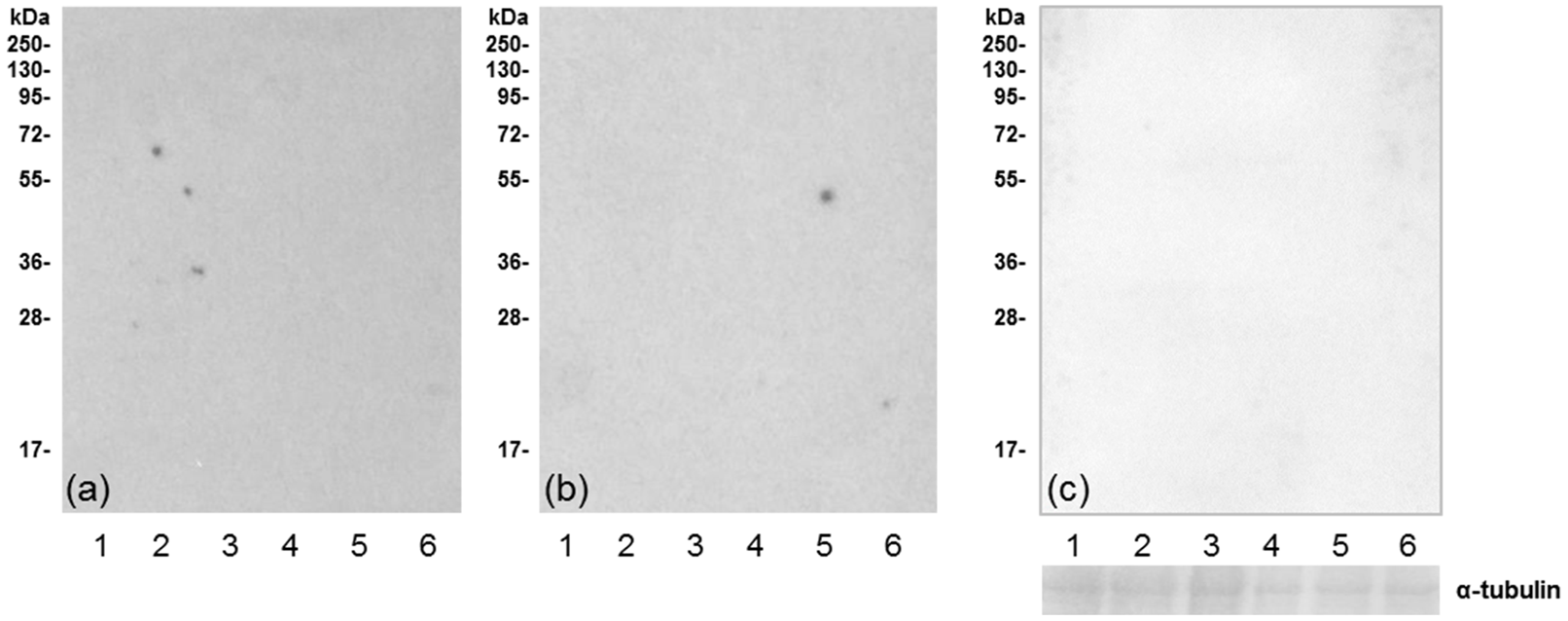

Supplement: Supplementary file 1 [file cells-09-00183-s001.zip › Supplement Figure S7.tif]
